# Online mapping of EMG signals into kinematics by autoencoding

**DOI:** 10.1186/s12984-018-0363-1

**Published:** 2018-03-13

**Authors:** Ivan Vujaklija, Vahid Shalchyan, Ernest N. Kamavuako, Ning Jiang, Hamid R. Marateb, Dario Farina

**Affiliations:** 1 Department of Bioengineering, Imperial College London, London, UK; 2 Biomedical Engineering Department, School of Electrical Engineering, Iran University of Science and Technology, Tehran, Iran; 3 Centre for Robotics Research, Department of Informatics, King’s College London, London, UK; 4 Department of Systems Design Engineering, University of Waterloo, Waterloo, ON Canada; 5 Biomedical Engineering Department, Engineering Faculty, University of Isfahan, Isfahan, Iran

**Keywords:** Prosthetic control, Myoelectric signal processing, Regression, Online performance, Autoencoding

## Abstract

**Background:**

In this paper, we propose a nonlinear minimally supervised method based on autoencoding (AEN) of EMG for myocontrol. The proposed method was tested against the state-of-the-art (SOA) control scheme using a Fitts’ law approach.

**Methods:**

Seven able-bodied subjects performed a series of target acquisition myoelectric control tasks using the AEN and SOA algorithms for controlling two degrees-of-freedom (radial/ulnar deviation and flexion/extension of the wrist), and their online performance was characterized by six metrics.

**Results:**

Both methods allowed a completion rate close to 100%, however AEN outperformed SOA for all other performance metrics, e.g. it allowed to perform the tasks on average in half the time with respect to SOA. Moreover, the amount of information transferred by the proposed method in bit/s was nearly twice the throughput of SOA.

**Conclusions:**

These results show that autoencoders can map EMG signals into kinematics with the potential of providing intuitive and dexterous control of artificial limbs for amputees.

## Background

Myoelectric signals (EMG) have been used to drive prosthetic devices for more than half a century. However, the commercially available products still mainly rely on a simple direct and sequential control. This control strategy offers robust and reliable handling of the prosthetic in daily life, but it allows limited recovery of functionality and requires high cognitive load by the user [1, 2]. Therefore, several attempts have been made for establishing a more intuitive interface for active prosthesis control.

Major advances in myocontrol have been made with pattern recognition approaches. These methods are based on the assumption that sufficiently distinguishable patterns can be observed in the EMG recordings during different motions. Each signal can be represented using a certain set of features which can be used as input to a classifier. The trained classifier is then capable of discriminating the intended motions. With state of the art pattern recognition methods, the classification accuracy exceeds > 95% when discriminating > 10 classes [3].

Despite their good performance and their recent translation in commercial systems [4], pattern recognition algorithms for myocontrol have some intrinsic limitations. For example, it is difficult to implement simultaneous and proportional control of multiple degrees of freedom (DoFs) with these algorithms since they do not allow a direct mapping of EMG into kinematics. This issue can be mitigated by the classification of complex movements as the combination of motions in combined classes [5–7], although this approach increases the training time and complexity.

More recently, regression approaches have been proposed for estimating the user activation intentions simultaneously and proportionally over multiple DoFs of wrist and hand [8–11]. When comparing linear and non-linear regression methods for myocontrol, differences were observed during offline processing [12]. However, in online tests, with the user in the loop, different regression methods performed similarly [13], indicating the important role of user adaptation to the interface. It has also been observed that regression allows a greater degree of user adaptation to the mapping as well as to signal non-stationarities than classic pattern recognition [14]. The high degree of user adaptation promoted by regression may allow the effective use of minimally supervised schemes. An example of these approaches is the factorization of the multi-channel EMG recordings with non-negative matrix factorization, which does not require labeling of the kinematics during training/calibration.

In this study, we propose and test a nonlinear mapping of EMG based on autoencoders (AEN), which exploits the advantage of unsupervised learning and the power of non-linear regression. AEN have been used in sleep analysis [15, 16], classification of arrhythmia [17], and detection of atrial fibrillation [18], as well as for the extraction of muscle synergies [19] and data compression [20]. Nevertheless, they have not been applied to myoelectric control. The aim of this study is therefore the development and validation against direct control of a method of autoencoding for proportional and simultaneous myocontrol.

## Methods

### Autoencoder

**Fig. 1:**
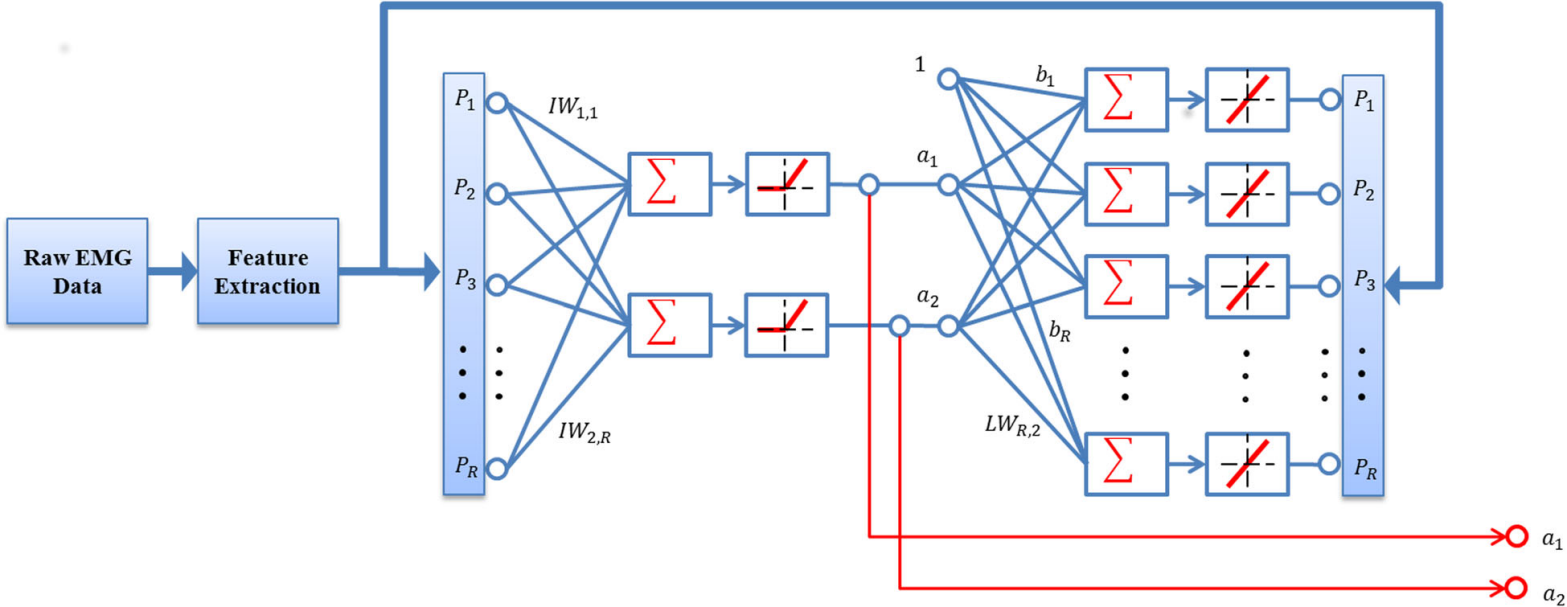
The structure of Autoencoder neural network for extracting control signals in two opposite directions (***a*1*,a*2**) for each DoF

Feed-forward neural networks, also referred to as multilayer perceptron (MLP), with one hidden layer have been widely used for learning continuous and bounded association functions between input data and a target output [21]. MLPs have also been previously used for estimating the kinematics of multiple DoFs from EMG features [9, 22]. In this study, we propose a different neural network approach for these estimates, based on AEN. Given the *R*-dimensional features of the surface EMG, denoted as *P*(*t*) = [*p*_1_(*t*), *p*_2_(*t*), ⋯, *p*_*R*_(*t*)], the goal is to estimate the activation intentions, or motor control signals, for each DoF. In this study, we used the root mean square (RMS) values of the *R*-channel surface EMG as *P*(*t*). The RMS values were obtained by non-overlapping 100 ms processing windows [23], which resulted in an output rate of the control of 10 Hz. The RMS feature vector was used simultaneously as both the input and the output of the AEN network, as shown in the structure of the network in Fig. 1.

The standard MLP structure with one hidden layer containing two neurons was used for each DoF for mapping the association of the EMG feature vector *P*(*t*) to itself, while capturing the low-dimensional controls in the hidden layer, with reduced number of neurons. To obtain the activation signals *a*_1_(*t*), *a*_2_(*t*) in both positive and negative directions at each DoF (i.e., flexion/extension or radial/ulnar deviation) in the hidden layer, two positive linear neurons with no biases from the input layer were used. This configuration was motivated by a physiological generative model based on muscle synergies [8] that suggests direction–wise estimation of DoF control. The output of the hidden layer is expressed as:

1 $$ \left[\begin{array}{c}{a}_1(t)\\ {}{a}_2(t)\end{array}\right]=\left| IW\bullet P(t)\right| $$

where |·| stands for the absolute value and *IW*(2 × *R*) represents the weights applied to the input layer in the network. The output layer has the same dimension as the input layer, and the transfer function of the output layer neurons was linear. The output of the network *Q*(*t*) is expressed as:

2 $$ Q(t)= LW\bullet a(t)+B $$

where *LW*(*R* × 2) represents the weight of the output layer and *B* represents the biases from the hidden neurons to the output neurons. The purpose of this network is to regenerate its input at the output, i.e. it imposes *Q*(*t*) ≈ *P*(*t*). However, the output is generated from a signal of reduced dimensionality with respect to the input and this lower-dimensional signal is used for control in this study.

For each DoF, the EMG feature vector was applied to the input and the output of an AEN with the structure shown in Fig. 1. The network was then trained using the Levenberg-Marquardt back-propagation algorithm, the combination of Gauss-Newton and the steepest descent methods [24]. The training was repeated 10 times with different initial weights, resulting in 10 AEN networks with different internal parameters for each DoF. Since the two outputs of the hidden layer estimate the activation of one DoF in opposite directions, the optimal set of network parameters was selected as that corresponding to the minimum correlation between the two outputs. Once the parameters were selected, the control signals $\left[{a}_1^i(t),{a}_2^i(t)\right]$for the *i*-th DoF could be extracted from each new feature vector by projection on the weight matrix *IW*^*i*^:

3 $$ \left[\begin{array}{c}{a}_1^i(t)\\ {}{a}_2^i(t)\end{array}\right]=\left|{IW}^i\bullet P(t)\right| $$

The indeterminacy of signal power for the activation signals was resolved by scaling them with correction factors *τ*_*ij*_ to obtain the final control signals with appropriate range of movement in the respective DoF:

4 $$ {c}^i(t)={\tau}_{i1}\bullet {a}_1^i(t)-{\tau}_{i2}\bullet {a}_2^i(t) $$

where the correction factors *τ*_*ij*_ were determined such that the resulting control signal *c*^*i*^(*t*) matched the range of movement in the *i*th DoFs, determined during the calibration phase (see Section IIC).

In the current application, two AEN networks were used to extract the activation signals corresponding to two DoFs (i.e., wrist flexion/extension and radial/ulnar deviation). The trained AEN input weight matrixes *IW*^*i*^ were used to control the DoFs simultaneously, according to Eqs. (3) and (4). The selected DoFs have been previously shown to have a fundamental functional relevance for patients [25]. Moreover, we limited the tests to 2 DoFs since the concurrent activation of 3 DoFs was challenging for most subjects. Therefore, the focus was on decoding and mapping flexion/extension and ulnar/radial deviation as representative wrist functions [12].

### Subjects

Seven able-bodied subjects without any neuromuscular disorders (5 M, 2 F, age: 29 ± 3 yrs) participated in the experiments. All subjects were presented with the detailed experiment protocol, which they had read and signed along with the informed consent approved by the research ethics committee of the University Medical Center Göttingen and conformed to the Declaration of Helsinki.

### Experimental protocol

The experimental protocol was similar to the one described in [23, 26]. Each participant was seated comfortably approximately 1 m in front of a computer screen, with the dominant arm extended at the side, fingers pointing towards the ground, and palm facing inside. Sixteen monopolar pre-gelled surface electrodes (Neuroline® 720, Ambu, Denmark) were placed around the forearm, with equal distance along the arm circumference. This resulted in two 8 electrode rings. The average inter-electrode distance within each ring was 23 mm, and the average distance between the rings was 20 mm. The electrode rings were mounted at a distance from the elbow of 1/3 of the length from the olecranon process to the styloid process of the ulna. The selected electrode arrangement provided EMG signals of high dimensionality, suited for the proposed analysis. Surface EMG was acquired by a commercial biosignal amplifier (EMGUSB2, OT Bioelettronica, Italy) at a sampling rate of 2048 Hz (12 bit A/D, 3 Hz to 900 Hz 6th-order Butterworth band-pass). A wrist band was used as reference electrode.

Other methods based on artificial neural networks required sample-by-sample labeled data with respect to joint kinematics for training (as in [22]). Conversely, in the current study, no kinematics was recorded. Rather, the intended activations at the multiple DoFs of each subject were estimated ‘blindly’ from the surface EMG.

#### Calibration phase

The calibration of the AEN relied solely on the EMG signals obtained during unconstrained, dynamic movements without kinematic labeling. The only restriction imposed during calibration was to articulate movements of one DoF at a time. Wrist flexion/extension (DoF1) and ulnar and radial deviation (DoF2) were the two DoFs used in this study. During calibration, for each DoF, six contractions covering the full range of motion were performed. These recordings were referred to as calibration contractions and their execution took approximately 3 min. The entire acquisition was made using a custom developed Matlab® program. Upon the successful calibration of the estimator, the online validation phase started.

#### Online validation phase

**Fig. 2:**
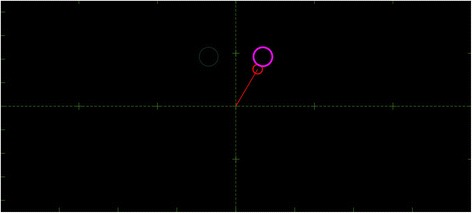
An example of virtual target scenario where the large circle on the right represents the currently active target and the smaller circle on the left is the next target. The red circle connected to the origin is the cursor controlled by the user which, during AEN-based control, moves vertically for ulnar/radial deviation and horizontally for wrist flexion/extension

Once the calibration phase was completed, the subjects were prompted to perform an online validation test. A cursor was presented on the user’s screen (Fig. 2). The 2-D displacements (horizontal and vertical) of the cursor were proportional to the wrist movements estimated from the surface EMG recordings in the two DoFs. Wrist flexion/extension movements were mapped to the horizontal displacement of the cursor, while wrist adduction/abduction (ulnar/radial deviations) movements were mapped to the vertical displacement. This virtual task paradigm is an adaptation of a system we have previously applied [23, 26, 27]. While there are more complex virtual environments to test myocontrol [28], this system offers an intuitive interface and associated quantitative evaluation metrics. The control was in position mode, i.e. when no EMG activity was detected the cursor returned to its original position at the origin of the workspace (at the center of the subject view). The correction factors (*τ*_*ij*_) were fine tuned for each subject to achieve effortless coverage of the full range of motion. This step took in total less than 1 min.

For the test phase, 20 circular targets were displayed in the subject view at predetermined positions (Fig. 7), one by one in a random order. The radius of each target was 8 density-independent pixels (dp), covering 0.7% of the entire workspace. On an audio cue, the subject was instructed to place the cursor within the circular targets and to keep it on the target for at least 300 ms for the task to be considered as successful. The dwelling time of 300 ms was chosen based on the previous work [23, 27], as a compromise between the functional evaluation and subject efforts, while at the same time preventing any bias towards either of the tested control strategies. The task execution time was limited to 20 s, after which the task was considered failed. The locations of the targets were chosen to ensure the need for activating all DoFs.

### Comparison with the state-of-the-art

In order to have a complete assessment of the capabilities of the proposed control algorithm, a comparison with the industrial state-of-the-art (SOA) control has been made. Therefore, the subjects repeated the same experiment using a direct control paradigm. The industrial SOA was implemented as standard sequential and proportional control requiring two bipolar measuring sites chosen so that a reliable one-site-one-function control could be achieved. For this control strategy, the experimenter chose the two bipolar derivations that led to the best control by the subject. Thresholds for each activation site were chosen using standard prosthesis fitting techniques, allowing easy control as well as the comfortable mode switch during co-contractions. For the SOA control scheme, the RMS values of the EMG signals were translated into the displacement velocity of the cursor, i.e. when the activation of one activation site passed the set threshold, the cursor moved along the mapped direction at a speed proportional to the RMS amplitude. When the EMG amplitude was below the threshold, the cursor would remain in its last position. This is the common control paradigm in commercial prostheses. While position control with the proposed technique required the concurrent activation of the DoFs to reach the targets, with velocity control all of them could essentially be completed with only sequential activations of the DoFs. Using position control for the SOA approach would have made the task completion impossible since the SOA approach does not allow simultaneous control. We chose to test the AEN with position control to specifically address the full potential of the new system. The same 20 targets were presented to the subjects for both control systems. Since the implementation of the SOA control was made in velocity mode, all the targets were reachable by switching between the two DoFs (sequential activations). The two control strategies were performed in random order between different subjects.

### Performance metrics

During each task execution, the trajectory of the cursor over time was recorded. The following six performance matrices were calculated in order to quantify the subject’s online control performance [29, 30]: Completion rate (in %) - the number of completed tasks over the total number of attempted tasks. Completion time (*t*_*c*_, [s]) - the average time it took the subject to complete the successful attempts. Overshoots - the number of incidents that the tip of the arrow passed through the target before the dwelling time was reached. Throughput (*TP*, [bit/s]) – the average ratio of the index of difficulty (*ID*) of each target and the completion time (CT). Speed (dp/s) - the ratio of the trajectory length formed by the center of the moving cursor (Fig. 2) and the completion time (*CT*). Path Efficiency (in %) - the ratio between the length of the optimal path from the initial point to the target and the actual trajectory realized [30] (a value of 100% indicates a perfect execution). The values of the controlled angles were mapped to the Cartesian coordinates.

The *throughput* (*TP*) is the amount of information that the user transmits through the interface and is defined as:

5 $$ TP=\frac{ID}{CT} $$

where *ID* is the task index of difficulty, and *t*_*c*_ is the task completion time. *TD* represents the Shannon’s extension of the Fitts’ law [29], as presented in [30]:

6 $$ ID={\mathit{\log}}_2\left(\frac{A}{W}+1\right) $$

where the target width *W* is the radius of the targets and the target amplitude *A* was defined as:

7 $$ A={\left(0.5{\gamma}_1+0.5{\gamma}_2\right)}^2 $$

where *γ*_1_ and *γ*_2_characterize the necessary angles which are needed to be reached with respect to the first and the second DoF. The value of *W* was fixed to 0.08 and the values of A were 0.8, 1.2 and 1.4. Using Eq. (6), the tested indices of difficulty were 3.4 for targets requiring small angle displacements along both DoFs, 4.0 for large displacements along only one DoF, and 4.2 for large displacements along both DoFs.

### Statistics

For each performance metric, a two-way repeated measures analysis of variance (ANOVA) was used with factors methods (AEN vs. SOA) and IDs in order to assess the performance of the proposed approach with respect to SOA. *P*-values less than 0.05 were considered significant and Bonferroni correction was applies for pairwise comparison for significant difference in IDs. Results are reported as mean ± standard error.

## Results

**Fig. 3:**
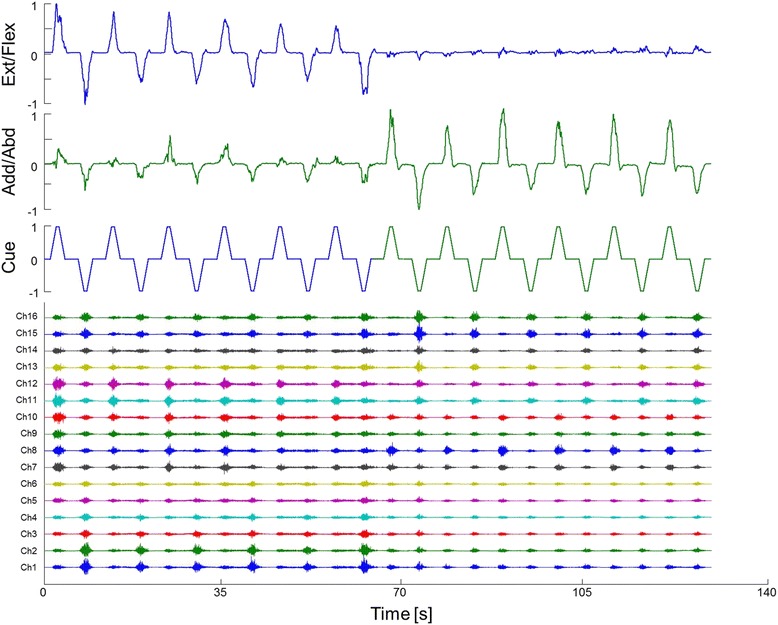
DoF wise mapping of the EMG signals (bottom traces) using the autoencoder for extension/flexion (upper trace) and adduction/abduction (middle trace)

During the calibration phase, AEN was used to estimate the output for each DoF. Figure 3 shows its performance for a representative subject.

**Fig. 4:**
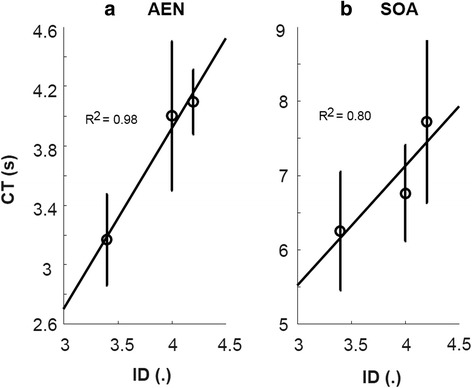
Linear relation between completion time (CT) on the vertical axis and index of difficulty (ID) on the horizontal axis for (**a**) autoencoder based control (AEN) and (**b**) state-of-the-art control scheme (SOA)

A strong linear relation was found between CT and ID for both control approaches, supporting the suitability of applying the Fitts’ Law test (Fig. 4).

**Fig. 5:**
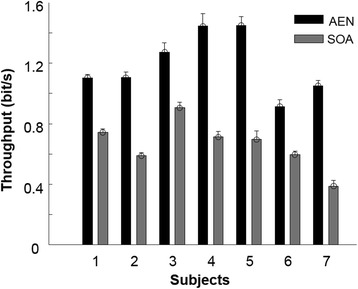
Throughput values for each subject

Subjects were able to complete nearly all the tasks with completion rate of 99.4 ± 0.06% and 100% for AEN and SOA, respectively (not significantly different). Completion time was significantly lower using AEN (3.75 ± 0.32 s) than SOA (6.91 ± 0.79 s) (*p* = 0.004). There was a significant difference between the first and third ID (*p* = 0.033) with no interaction (*p* = 0.295). A significant difference (*p* < 0.001) in Throughput was found between AEN (1.19 ± 0.08 bit/s) and SOA (0.66 ± 0.06 bit/s). The throughput for each subject is shown in Fig. 5.

**Fig. 6:**
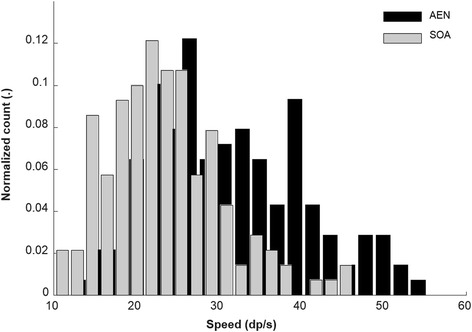
Distribution of speed across all targets and all subjects

AEN showed significantly higher (*p* = 0.029) Speed than SOA, with associated significant difference in ID, but without interaction (*p* = 0.590). This implies that the difference in Speed did not depend on the difficulty of the task. Figure 6 depicts the distribution of speed for both control schemes.

**Fig. 7:**
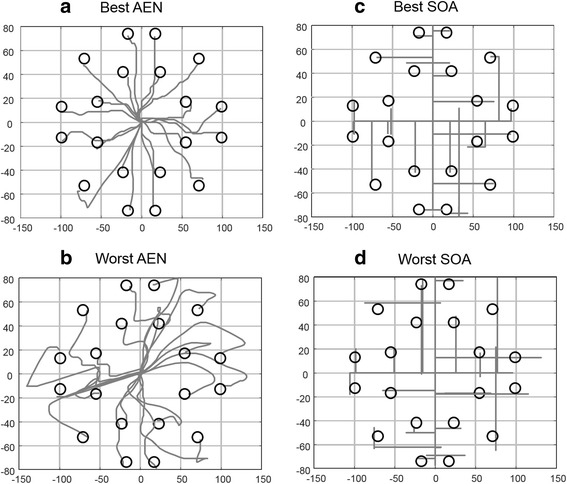
Visualization of best and worst path efficiencies with all targets for (**a** and **b**) autoencoder (AEN), (**c** and **d**) state-of-the-art (SOA)

Path efficiency was better (*p* < 0.001) using AEN (73.80 ± 4.98%) than SOA (54.10 ± 2.47%). The number of overshoots was significantly lower when using AEN than SOA (*p* = 0.008). Figure 7 shows an example of the best and worst path efficiencies for the AEN and SOA methods for all targets, showing the users activating both DoFs to reach the targets.

## Discussions

We proposed a minimally supervised method for EMG mapping based on autoencoding and tested it in online tasks against direct sequential control (i.e. SOA) using a Fitts’ law approach. Both methods allowed a completion rate close to 100%, however, AEN outperformed SOA for all other performance metrics, e.g. it allowed to perform the tasks on average in half the time with respect to SOA.

The performance of the proposed method is similar to that of previous studies using non-negative factorization, linear regression and artificial neural networks, with [22, 31] and without [26, 32, 33] the use of kinematics labeling during training. The performance of SOA in this study was also similar to previously reported results [23] for completion rate and completion time. Moreover, we observed a skewness of the distribution of speeds with SOA towards low values.

Previously proposed methods for EMG mapping that do not require supervised training are based on signal factorization. Here we presented a different approach that projects the EMG signals into a lower-dimensional space for control based on autoencoding. This method requires a brief calibration procedure during which the user performs single DoF contractions only prompted by the visual cue and without any additional kinematic recordings. Therefore, the mapping does not require amputee users to perform bilateral motions in order to obtain kinetic or kinematic labels from the contralateral limb [9, 22, 34]. In addition, this makes the system fully applicable even in the case of bilateral amputees. In the proposed method, the calibration provides estimates of the projecting matrices by two AEN (one for each DoF) which are then used concurrently for simultaneous and proportional control. The amount of information transmitted by the proposed method was almost twice the throughput of SOA.

The possibility to train without kinematic recordings makes the system practical in clinical applications. On the other hand, it makes the assumption of a linear association between primitives extracted from the EMG and kinematics. Nonetheless, even if this assumption is not exactly met, reliable simultaneous and proportional myoelectric control does not require a high accuracy in the mapping between EMG and kinematics during online control. Indeed, accurate online myoelectric control can be achieved by the continuous interaction and adaptation of the user to the myoelectric controller [13].

This feasibility study allowed rigorous testing of the proposed concept in a controlled laboratory environment that showed high potential of the approach. Future efforts should focus on implementing a full clinical assessment on patients.

## Conclusions

We presented a new approach for mapping EMG signals into commands for multiple degrees of freedom to achieve simultaneous and proportional control. The method has been validated in online tests in a group of able-bodied individuals and has shown high transfer rate with respect to direct SOA control.

## Abbreviations

AEN: Autoencoders
CR: Completion Rate
CT: Completion time
DoF: Degree-of-freedom
EMG: Electromyogram
ID: Index of difficulty
RMS: Root Mean Square
SOA: State-of-the-art
TP: Throughput
